# Clinical Effects and Safety of Auricular Acupressure as an Adjunct Therapy on Postoperative Pain among Patients with Hip Fracture: A Meta-Analysis

**DOI:** 10.1155/2023/5077772

**Published:** 2023-04-24

**Authors:** Pin Li, Hongyun Chen, Xiuzhen Fu, Haili Zhou, Fan Lai

**Affiliations:** Department of Orthopedic, The Second Affiliated Hospital of Guangzhou University of Chinese Medicine, Guangzhou, China

## Abstract

**Objectives:**

To evaluate the short-term outcome of treatment by auricular acupressure (AA) on postoperative pain among hip fracture (HF) patients.

**Methods:**

A systematic search for randomized controlled trials on this topic was conducted through May 2022 by searching multiple English and Chinese databases. The methodological quality of the included trails was assessed by the Cochrane Handbook tool, and relevant data were extracted and statistically analyzed by RevMan 5.4.1 software. The quality of the evidence supporting each outcome was evaluated by GRADEpro GDT.

**Results:**

Fourteen trials with a total of 1390 participants were included in this study. Compared with conventional treatment (CT) alone, the combination of AA and CT had a significantly greater effect on the visual analog scale at 12 h (MD −0.53, 95% CI −0.77 to −0.30), 24 h (MD −0.59, 95% CI −0.92 to −0.25), 36 h (MD −0.07, 95% CI −0.13 to −0.02), 48 h (MD −0.52, 95% CI −0.97 to −0.08), and 72 h (MD −0.72, 95% CI −1.02 to −0.42), amount of analgesics used (MD −12.35, 95% CI −14.21 to −10.48), Harris Hip Score (MD 6.58, 95% CI 3.60 to 9.56), effective rate (OR 6.37, 95% CI 2.68 to 15.15), and adverse events (OR 0.35, 95% CI 0.17 to 0.71).

**Conclusions:**

Compared with CT alone, the combination of AA and CRT had a significantly greater effect on postoperative pain in HF patients. However, trails with a rigorous methodology, including standard protocols for AA and multiethnic subjects, are still needed.

## 1. Introduction

As the aging process of the population continues to accelerate, the proportion of the elderly (>60 years) will continue to increase [1]. It is estimated that by 2050, the proportion of the elderly population will reach 21.1% worldwide [2, 3]. Hip fracture is a common type of fracture in the elderly and ranks among the top 10 of disability [4]. It is estimated that the absolute number of hip fractures is expected to increase from 1.6 million in 2000 to 6.3 million by the year 2050 [5]. Hip fracture (HF) has become a worldwide health problem, it is estimated that the annual cost of HF treatment has increased from approximately 10.3 to 15.2 billion dollars in 1990 to 17 billion in 2002 [6].

Timely surgery for hip fractures remains the mainstay of treatment, including internal fixation, total hip arthroplasty, and hemiarthroplasty [7]. Many official clinical societies recommend postoperative multi‐modal analgesia [8] because elderly patients with inadequate postoperative pain control are reluctant to mobilize, thus increasing the potential risk of complications and slowing recovery [9]. Adequate analgesia is of great significance. Current strategies for pain management include oral and parenteral systemic analgesia, and systematic administration of opioids remains the most commonly used analgesia protocol [10]. While opioids are effective in relieving static pain, they may not be sufficient for dynamic pain [11]. Furthermore, the use of opioids may bring side effects, such as delirium, drowsiness, and even respiratory depression, which may affect the prognosis of patients [12]. Thus, to lower the risk of adverse events and also guarantee treatment efficacy, complementary and alternative therapies have been investigated and compared.

Acupuncture is a traditional nonpharmacological treatment in China and has been widely recognized worldwide [13]. Available evidence suggests that acupuncture is effective for pain relief, thus the World Health Organization recommends the use of acupuncture for a variety of pains, including postoperative pain [13]. As an important component of acupuncture [14], auricular acupressure (AA) has also been deemed effective for pain management by the National Institutes of Health [15]. A literature search yielded many published clinical randomized controlled trials (RCTs) of AA for postoperative pain among HF patients. Consequently, the aim of this study was to evaluate the short-term outcome of treatment by AA on postoperative pain among HF patients by conducting a systematic literature review and meta-analysis of RCTs.

## 2. Methods

The protocol of this study was registered in PROSPERO (https://www.crd.York.ac.uk/prospero). This work was performed following criteria in the Cochrane Handbook [16] and reported in line with preferred reporting items for systematic reviews and meta-analyses (PRISMA) [17].

### 2.1. Search Strategy

Eight databases (Pubmed, EMBASE, Cochrane Library, Web of Science, China National Knowledge, and Wan Fang Database, China National Knowledge Infrastructure, Chongqing VIP, and Sino-Med) were searched on May 18, 2022, using the following keywords: auricular acupressure, hip fracture, randomized clinical trials. The detailed search strategy for PubMed is given in supplementary file A.

### 2.2. Criteria for Considering Studies

Included criteria were as follows: (i) type of study: published randomized controlled trial (RCT) in English and Chinese; (ii) intervention: AA with conventional treatment (CT); (iii) comparison: CT; (iv) population: diagnosed as having a hip fracture confirmed by imaging, regardless of race, sex, or age; and (v) outcome: pain intensity (visual analog scale (VAS)) at 12, 24, 36, 48, and 72 h after surgery the amount of analgesics used, the Harris Hip Score (HPS), the effective rate (ER), and adverse events (AE). In general, ER is defined by the formula: ER =  (“total number of patients” − “number of patients without response”)/total number of patients; “no response” is defined as no significant change in VAS score after treatment. Trails of AA with more than one Traditional Chinese medicine treatment technique as an intervention were also excluded.

### 2.3. Study Identification

Search results were imported into Endnote and duplicates were removed. Two research studies independently screen the titles and abstracts of the retrieved articles, evaluate the potential full texts, and determine the eligibility of the reviews. Any discrepancies were solved by introducing a third researcher for judgment.

Data were extracted by two independent research studies using a predefined form, including: first author, year, country, simple size, characteristics of patients, course of disease, treatment protocol, outcome indicators, and consequences of and outcomes. Resolve any discrepancies through consultation.

### 2.4. Quality Assessment

Two research studies independently assessed the risk of bias by using the Cochrane Collaboration tool [18]. Each trail could be judged to be at “low,” “high,” or “unclear” risk of bias according to the domains of random sequence generation, allocation concealment, blinding of participants and personnel, blinding of outcome assessment, incomplete dataset, selective reporting, and other bias. If more than half of the domains were assessed as having a low risk of bias, the trial was assessed as having a low overall risk of bias; if more than half of the domains were assessed as having a high risk of bias or an unclear risk of bias, the trial was assessed as having a high overall risk of bias. Resolve any discrepancies through consultation.

### 2.5. Data Synthesis

The odds ratio (OR) with 95% of CIs for dichotomous outcomes and the mean difference (MD) for continuous variables. The heterogeneity of the studies was assessed using the *I*^2^. If *I*^2^ < 50%, there was no significant heterogeneity among studies, and a fixed effect model was used to analyze the data. If *I*^2^ ≥ 50% of the heterogeneity among the studies was significant and a random effect model was used to provide the evaluations of the intervention. Subgroup analyses were determined by whether the obtained data were sufficient. A funnel plot was used to assess publication bias.

### 2.6. Level of Evidence

Two research studies independently assessed the certainty of evidence for each outcome using a grading of recommendations assessment, development, and evaluation (GRADE) system [19]. The GRADE guideline consists of seven domains, namely, risk of bias, inconsistency, indirectness, imprecision, and publication bias. The certainty of the evidence was rated as high, moderate, low, and very low. The summary of findings table was created by the GRADEpro GDT (https://gradepro.org/). Resolve any discrepancies through consultation.

## 3. Results

### 3.1. Literature Screening

**Figure 1:**
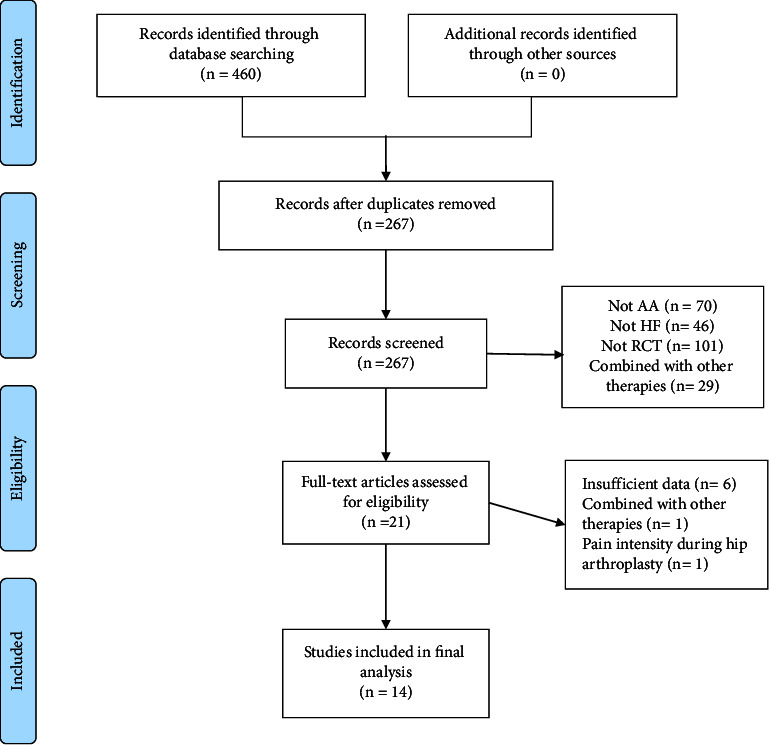
Literature screening flowchart.

Literature screening flowchart is shown in Figure 1. A total of 460 trails were identified. After removing duplications, the titles and abstracts of 267 trails were further evaluated. Next, the full texts of the remaining 21 records were assessed, and 14 trails were finalized for inclusion in our meta-analysis [20–33].

### 3.2. General Characteristics

Characteristics of the included trails are presented in Table 1. 1390 participants were included from the 14 RCTs, which were published between 2012 and 2022. All of them were conducted in China and reported nonsignificant differences in their patient baseline characteristics. The main assessment tools were the VAS and the amount of analgesics used, HPS: Harris Hip Score, ER, and AE.

### 3.3. Methodological Quality Assessment

**Figure 2:**
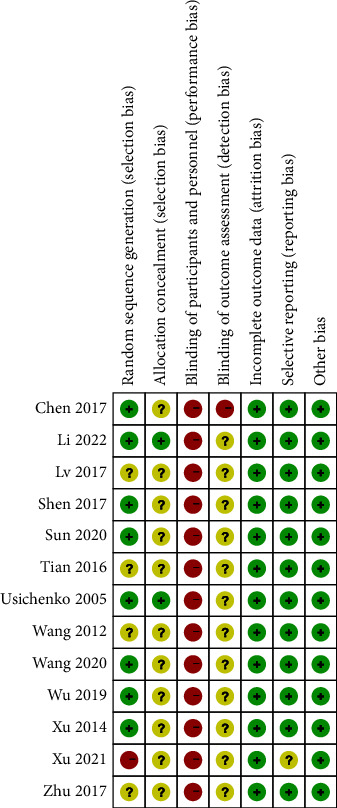
Risk of bias summary.

**Figure 3:**
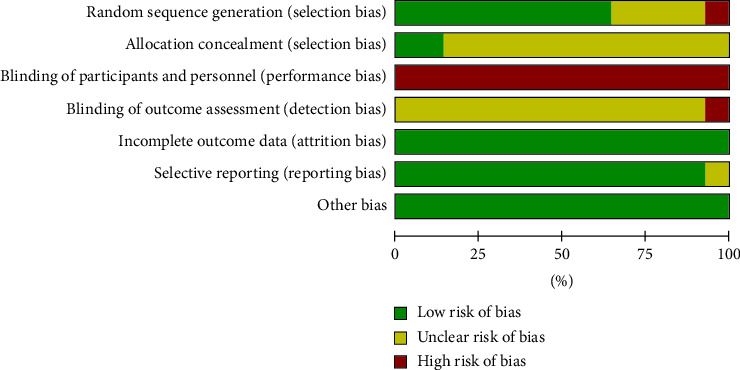
Risk of bias graph.

As shown in Figures 2 and 3, the risk of bias in the included studies was mainly derived from random sequence generation, allocation concealment, blinding of participants and personnel, and blinding of outcome assessment. Finally, 8 of the 14 RCTs were assessed as having a low overall risk of bias, and the rest had a high overall risk of bias.

### 3.4. Results of Meta-Analyses

#### 3.4.1. Visual Analog Scale

**Figure 4:**
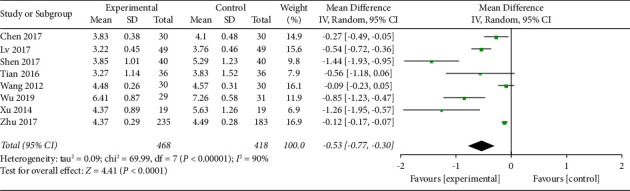
Comparison of the VAS at 12 h between the AA group and the control group.

*(1) Visual Analog Scale at 12 h*. VAS was used to score the pain degree of patients, which the score was in direct proportion to the pain degree. Eight trials with a total of 886 participants recorded VAS at 12 h of the intervention. The random effect model was applied, and pool results showed that AA could reduce VAS significantly in the experimental group than control group (MD −0.53, 95% CI −0.77 to −0.30) as shown in Figure 4. In addition, the data provided by the included studies were insufficient to support subgroup analyses, and therefore no further subgroup analyses were performed in subsequent analyses. The funnel plot was given in supplementary file B.

**Figure 5:**
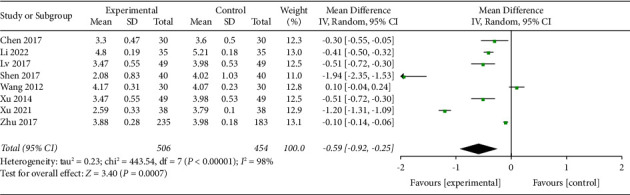
Comparison of the VAS at 24 h between the AA group and the control group.

*(2) Visual Analog Scale at 24 h*. Eight trials with a total of 960 participants recorded VAS at 24 h of the intervention. The random effect model was applied, and pool results showed that AA could reduce VAS significantly in the experimental group than the control group (MD −0.59, 95% CI −0.92 to −0.25) as shown in Figure 5. Funnel plot was given in supplementary file B.

**Figure 6:**
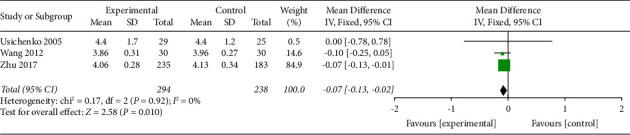
Comparison of the VAS at 36 h between the AA group and the control group.

*(3) Visual Analog Scale at 36 h*. Three trials with a total of 532 participants recorded VAS at 36 h of the intervention. The fixed effect model was applied, and pool results showed that AA could reduce VAS significantly in experimental group than the control group (MD −0.07, 95% CI −0.13 to −0.02) as shown in Figure 6. Funnel plot was given in supplementary file B.

**Figure 7:**
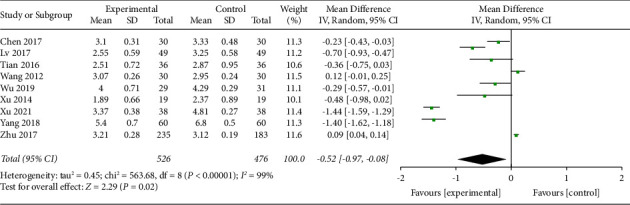
Comparison of the VAS at 48 h between the AA group and the control group.

*(4) Visual Analog Scale at 48 h*. Nine trials with a total of 1002 participants recorded VAS at 48 h of the intervention. The random effect model was applied, and pool results showed that AA could reduce VAS significantly in the experimental group than control group (MD −0.52, 95% CI −0.97 to −0.08) as shown in Figure 7. The funnel plot was given in supplementary file B.

**Figure 8:**
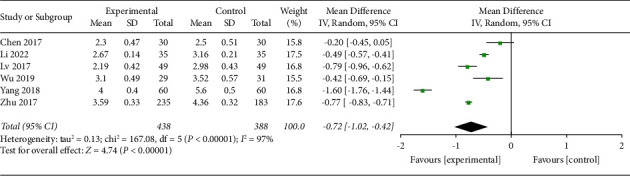
Comparison of the VAS at 72 h between the AA group and the control group.

*(5) Visual Analog Scale at 72 h*. Six trials with a total of 826 participants recorded VAS at 72 h of the intervention. The random effect model was applied, pool results showed that AA could reduce VAS significantly in the experimental group than control group (MD −0.72, 95% CI −1.02 to −0.42), as shown in Figure 8. The funnel plot was given in supplementary file B.

#### 3.4.2. Harris Hip Score

**Figure 9:**
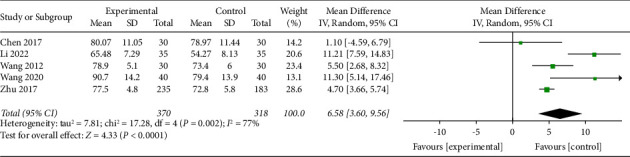
Comparison of the HHS between the AA group and the control group.

Five trials with a total of 688 participants recorded HHS at the end of the intervention. The random effect model was applied and the pool results showed that AA could improve HHS significantly in the experimental group than control group (MD 6.58, 95% CI 3.60 to 9.56), as shown in Figure 9. The funnel plot was given in supplementary file B.

#### 3.4.3. Amount of Analgesics Used

**Figure 10:**
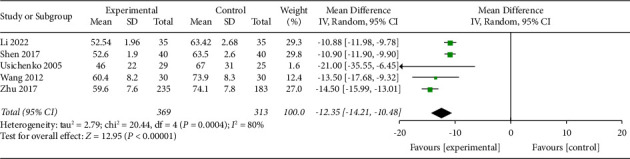
Comparison of the amount of analgesics used between the AA group and the control group.

Five trials with a total of 682 participants recorded the amount of analgesics used at the end of the intervention. The random effects model was applied, and pool results showed that AA could reduce the amount of analgesics used significantly more in the experimental group than the control group (MD −12.35, 95% CI −14.21 to −10.48), as shown in Figure 10. The funnel plot was given in supplementary file B.

#### 3.4.4. Effective Rate

**Figure 11:**
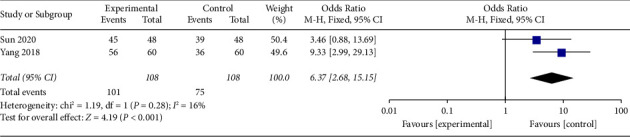
Comparison of the effective rate between the AA group and the control group.

Two trials, with a total of 176 participants, recorded an effective rate at the end of the intervention. The fixed effect model was applied, and pool results showed that AA could increase the effective rate significantly in the experimental group than control group (OR 6.37, 95% CI 2.68 to 15.15), as shown in Figure 11. The funnel plot was given in supplementary file B.

#### 3.4.5. Adverse Events

**Figure 12:**
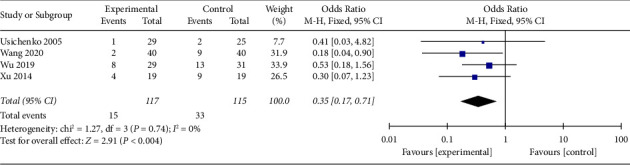
Comparison of the adverse events between the AA group and the control group.

Four trials with a total of 232 participants recorded adverse events at the end of the intervention. The fixed effect model was applied, pool results showed that AA could reduce adverse events significantly in experimental group than control group (OR 0.35, 95% CI 0.17 to 0.71), as shown in Figure 12. Funnel plot was given in supplementary file B.

### 3.5. Evidence Quality Assessment

Due to limitations of the enrolled trails, the strength of the evidence was weakened for all outcomes. Inconsistency, imprecision, and publication bias also limited the strength of the evidence for some outcomes.

Finally, one of the outcomes was assessed as low moderate quality, and the rest were of low or very low quality. Funnel graphs for these outcomes are given in Appendix B. Details are outlined in Table 2.

## 4. Discussion

In recent years, there have been more publications on AA for postoperative pain among patients with HF. However, interpretation of this evidence from these studies is difficult. We conducted this study to systematically evaluate the clinical effect of AA on postoperative pain in HF patients.

### 4.1. Summary of Main Results

In this study, 14 trails involving 1390 patients were included for meta-analysis. First, the pooled results suggested that AA combined with CT was significantly superior to CT alone in terms of VAS at 12, 24, 48, 36 h, and 72 h, HHS, amount of analgesics used, ER, and AE. These results indicated that AA can help reduce postoperative pain degree, reduce the amount of analgesics, improve hip function, and reduce the incidence of AE among HF patients. Second, it should be emphasized that methodological flaws are prevalent in existing RCTs. Most RCTs did not report proper allocation concealment or blinding procedures for outcome assessments. Furthermore, no study was blinded to participants and personnel, suggesting potential performance bias. Of the 7 bias items, only one study met the requirement for low risk of bias. Third, the evidence quality was generally evaluated as “moderate” “low” or “very low” by the GRADE system. No study was rated as having a high level of evidence. Although AA has been widely used in China, and the pooled analysis of this study has yielded that AA combined with CT was likely to have potential therapeutic benefits in postoperative pain among HF patients, but the level of evidence was not high. There may still be gaps between the evidence supporting the efficacy of AA and its clinical implementation. Further trials with rigorous methodology, including standard protocols for AA and multiethnic subjects, are still warranted to provide stronger evidence. Fourth, limited by insufficient data, this study was unable to evaluate the long-term effects of AA. The included studies only evaluated the use of AA for several days and assessed outcomes before and immediately after treatment, therefore, the long-term effects of AA on postoperative pain could not be revealed.

### 4.2. Agreements and Disagreements with Other Studies or Reviews

This review agrees with the results of the other study [34] in the aspect that AA as a complement to conventional drugs reduces postoperative pain, though with uncertainty. A systematic review [34] reported that in postoperative patients with fractures treated with AA, the degree of pain significantly improved 24 hours after surgery and was also significantly lower than in the control group in terms of ER. These results agree with our study. However, this review [34] focused only on pain at one time point and did not address the question of duration of action, and this study included patients with all types of fractures. The difference in our review is that we focused on patients with HF and observed pain at multiple time points after surgery and also on HHS, amount of analgesics used, and AE, making the assessment more comprehensive. In addition, as in the previous review, this study was also limited by the high risk of bias in the included trials, so the level of evidence obtained was not high. More well-designed, rigorous, and large trials are needed in this field.

### 4.3. Implications for Practice

In addition to shorter hospital stays and reduced morbidity and mortality, effective relief of acute postoperative pain is associated with increased patient satisfaction [33]. AA is an ancient Chinese nonpharmacological treatment that has been reported to be effective and safe in improving multiple factors in fracture patients and has the potential to promote postoperative recovery in combination with CT. Although the current review did not provide the best evidence, the results suggested that the combination of AA with CT postoperative pain degree, reduce the amount of analgesics, improve hip function, and reduce the incidence of AE among HF patients.

AA has been used to treat various types of pain, including postoperative, musculoskeletal pain, and pain associated with anesthesia [35]. The analgesic effect of AA has been preliminarily revealed. Research studies have found that AA stimulation can activate the descending pain inhibitory pathway in the brainstem-spinal cord and inhibit the ascending pain pathway, which in turn exerts analgesic effects [36]. It has been found that acupoint stimulation of one or both ears can increase the pain threshold [37], and this effect peaks 5–10 minutes after stimulation and lasts for hours to days [38]. Furthermore, it has been suggested that the analgesic effect of AA is also associated with the endogenous opioid system [39].

### 4.4. Limitations

Considering that all included trails were conducted in China, it should be noted that publication bias was also observed in this meta-analysis, which indicates that the results of this review may be challenging to generalize, especially in countries other than China. As a country that has practiced AA for a long time, Chinese attitudes towards AA may be more favorable than those of other ethnic groups, which may contribute to the placebo effect. Therefore, further studies in countries other than China are still needed. Furthermore, the diversity of the AA protocol used in the included trails may contribute to the heterogeneity of the findings [40]. While complementary and alternative therapies, such as AA, may emphasize tailoring treatments to individual patient characteristics, developing basic treatment standards that allow for some modifications can improve the quality of clinical evidence in this field.

## 5. Conclusion

Compared with CT alone, the combination of AA and CRT had a significantly greater effect on postoperative pain in HF patients. However, trails with a rigorous methodology, including standard protocols for AA and multiethnic subjects, are still needed.

## Figures and Tables

**Table 1:** Characteristics of the included trails.

Study	Simple size	Age		Intervention		Therapy duration	Outcomes
		*I*	*C*	*I*	*C*		
Li et al. [20]	35/35	68.94 ± 5.66	69.67 ± 5.52	AA + CT	CT	3∼5 t/d, 3 d	VAS, HHS, AAS
Xu and Li [21]	38/38	66.80 ± 3.73	66.88 ± 3.79	AA + CT	CT	3∼5 t/d, 2 d	VAS, AAS
Sun [22]	48/48	N/A	N/A	AA + CT	CT	3∼5 t/d, 3 d	ER
Wu and Wang [23]	35/33	39.5 ± 7.1	41.3 ± 7.2	AA + CT	CT	4∼6 t/d, 3 d	VAS, AAS, AE
Wang et al. [24]	40/40	N/A	N/A	AA + CT	CT	3∼5 t/d	HHS, AE
Yang [25]	60/60	54.6 ± 8.1	53.4 ± 8.3	AA + CT	CT	3∼4 t/d	VAS, ER
Chen [26]	30/30	73.56 ± 7.09	73.48 ± 6.82	AA + CT	CT	4 t/d	VAS
Lv [27]	49/49	59.8 ± 8.6	59.5 ± 8.7	AA + CT	CT	3∼5 t/d, 3 d	VAS
Zhu [28]	235/183	59.84 ± 6.13	60.19 ± 5.74	AA + CT	CT	4 t/d	VAS, HHS, AAS, AE
Shen and Zhou [29]	40/40	N/A	N/A	AA + CT	CT	3∼5 t/d, 3 d	VAS, HHS
Tian et al. [30]	36/36	N/A	N/A	AA + CT	CT	3∼5 t/d, 3 d	VAS
Xu [31]	19/19	60.74 ± 8.76	59.32 ± 7.68	AA + CT	CT	3∼5 t/d, 3 d	VAS, AE
Wang et al. [32]	30/30	60.93 ± 5.90	59.87 ± 6.21	AA + CT	CT	4 t/d	VAS, HHS, AAS, AE
Usichenko et al. [33]	29/25	68 ± 10	66 ± 11	AA + CT	CT	3 d	VAS, AAS, AE

*C*: control group; *I*: intervention group; N/A: not applicable AA: auricular acupressure; CT: conventional treatment; VAS: visual analog scale; AAS: amount of analgesics used; HHS: Harris Hip Score; ER: effective rate; AE: adverse events.

**Table 2:** Level of evidence.

Outcomes		Certainty assessment						Mean difference (95% CI)	Certainty of the evidence (GRADE)
		No of participants (studies)	Limitations	Inconsistency	Indirectness	Imprecision	Publication bias		
VAS	12 h	886 (8)	Serious^①^	Serious^②^	No	No	Serious^④^	MD−0.53, 95% CI −0.77 to −0.30	⨁⨁◯◯◯ very low
	24 h	960 (8)	Serious^①^	Serious^②^	No	No	Serious^④^	MD−0.59, 95% CI −0.92 to −0.25	⨁⨁◯◯◯ very low
	36 h	532 (3)	Serious^①^	No	No	No	No	MD −0.07, 95% CI −0.13 to −0.02	⨁⨁⨁⨁◯ Moderate
	48 h	9 (1002)	Serious^①^	Serious^②^	No	No	Serious^④^	MD −0.52, 95% CI −0.97 to −0.08	⨁⨁◯◯◯ very low
	72 h	6 (826)	Serious^①^	Serious^②^	No	No	Serious^④^	MD −0.72, 95% CI −1.02 to −0.42	⨁⨁◯◯◯ very low

AAS		4 (682)	Serious^①^	Serious^②^	No	No	Serious^④^	MD −12.35, 95% CI−14.21 to −10.48	⨁⨁◯◯◯ very low

HHS		5 (688)	Serious^①^	Serious^②^	No	No	Serious^④^	MD 6.58, 95% CI 3.60 to 9.56	⨁⨁◯◯◯ Very low

ER		2 (176)	Serious^①^	No	No	Serious^③^	Serious^④^	OR 6.37, 95% CI 2.68 to 15.15	⨁⨁⨁◯◯ Low

AE		4 (232)	Serious^①^	No	No	No	Serious^④^	OR 0.35, 95% CI 0.17 to 0.71	⨁⨁⨁◯◯ Low

OR: odds ratio; MD: MD: mean difference; VAS: visual analog scale; AAS: amount of analgesics used; HHS: Harris Hip Score; ER: effective rate; AE: adverse events; ^①^the experimental design had a large bias in its random, distributive findings or was blinded; ^②^the confidence interval overlapped less, the *P* value of the heterogeneity test was very small, and the *I*^2^ was larger; ^③^the confidence interval was not narrow enough, or the simple size was small; ^④^funnel graph asymmetry.

## Data Availability

The detailed search strategy is given in Appendix A. All analyses were based on previously published studies.

## Abbreviations

AA: Auricular acupressure
HF: Hip fracture
N/A: Not applicable
AA: Auricular acupressure
CT: Conventional treatment
VAS: Visual analog scale
AAS: Amount of analgesics used
HHS: Harris Hip Score
ER: Effective rate
AE: Adverse events
GRADE: Grading of recommendations, assessment, development, and evaluation
RCTs: Randomized clinical trials
PRISMA: Preferred reporting items for systematic reviews and meta-analyses
OR: Odds ratio
SMD: Standardized mean difference.

## Supplementary Materials

Supplementary Materials The detailed search strategy for the PubMed is given in supplementary file A. Funnel plot for outcomes was given in supplementary file B.
