# Supplementary material for: Clinical Effects and Safety of Auricular Acupressure as an Adjunct Therapy on Postoperative Pain among Patients with Hip Fracture: A Meta-Analysis
Source: Pain Res Manag. 2023 Apr 24;2023:5077772. doi: 10.1155/2023/5077772 (PMC10151718; doi:10.1155/2023/5077772)
Supplement: Supplementary Materials — The detailed search strategy for the PubMed is given in supplementary file A. Funnel plot for outcomes was given in supplementary file B. [file 5077772.f1.docx]

**Supplementary file A**: **The search strategy for PubMed**

#1 "Hip fractures"[Mesh]

#2 Hip fractures[Title/Abstract] OR trochanteric fractures[Title/Abstract] OR intertrochanteric fractures[Title/Abstract] OR subtrochanteric fractures[Title/Abstract] OR hip surgery[Title/Abstract] OR femur fracture[Title/Abstract] OR femur neck fracture[Title/Abstract]

#3 #1 OR #2

#4 "auricular acupuncture"[Mesh] OR "ear acupuncture"[Mesh]

#5 auricular acupuncture [Title/Abstract] OR ear acupuncture[Title/Abstract] OR auricular acupressure[Title/Abstract] OR auricular therapy[Title/Abstract] OR acupressur* [Title/Abstract] OR auricular[Title/Abstract] OR auriculotherap*[Title/Abstract] OR auriculotherapy[Title/Abstract] OR auricular needle[Title/Abstract]

#6 #4 OR #5

**Supplementary file B:** **Funnel plot for outcomes**


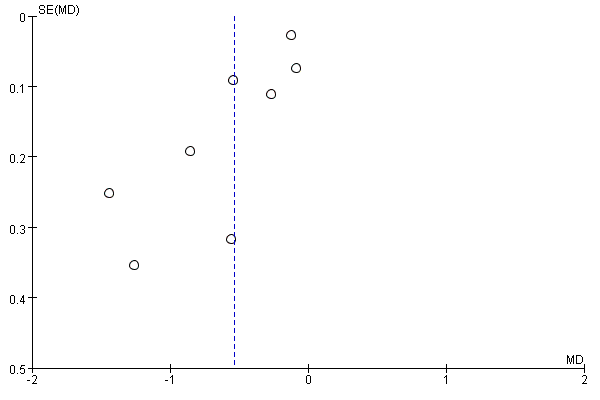


Figure 1. Funnel plot of the VAS at 12 h.


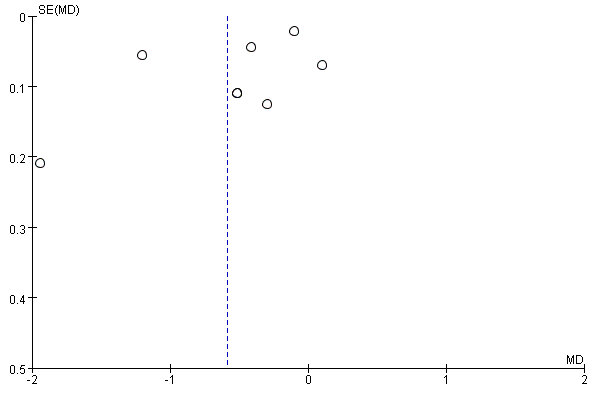


Figure 2. Funnel plot of the VAS at 24 h.


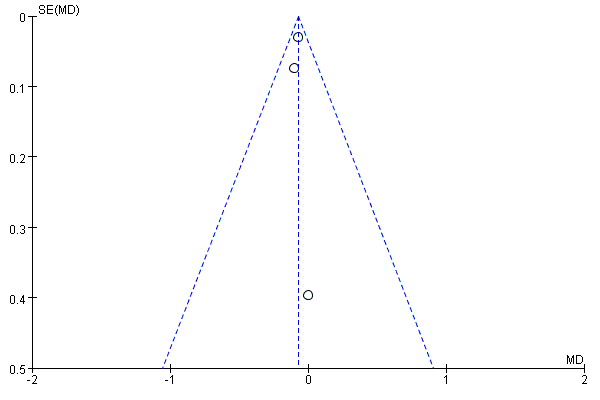


Figure 3. Funnel plot of the VAS at 36 h.


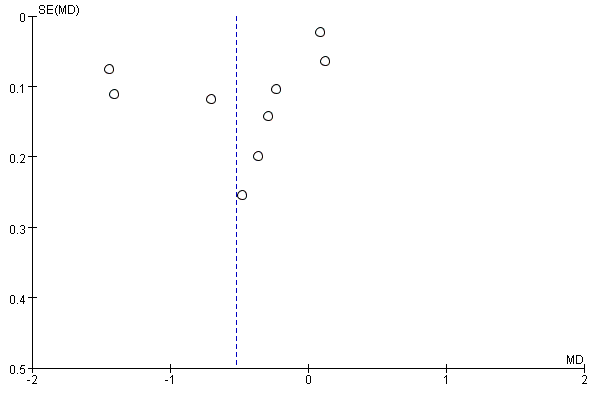


Figure 4. Funnel plot of the VAS at 48 h.


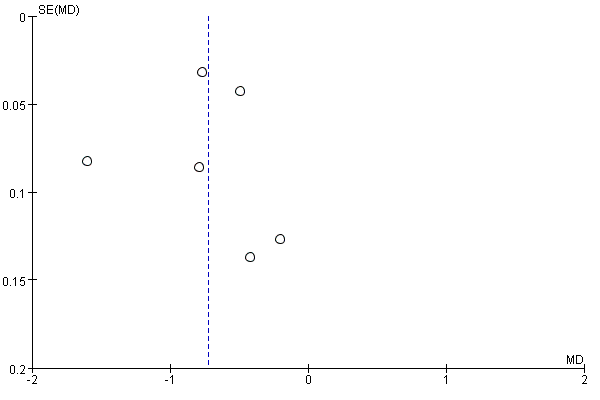


Figure 5. Funnel plot of the VAS at 72 h.

.


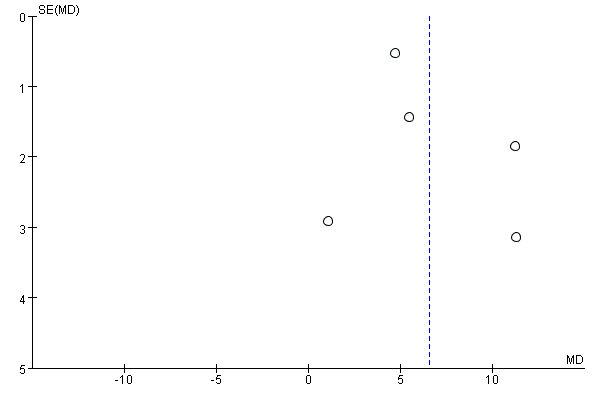


Figure 6. Funnel plot of the Harris hip score.


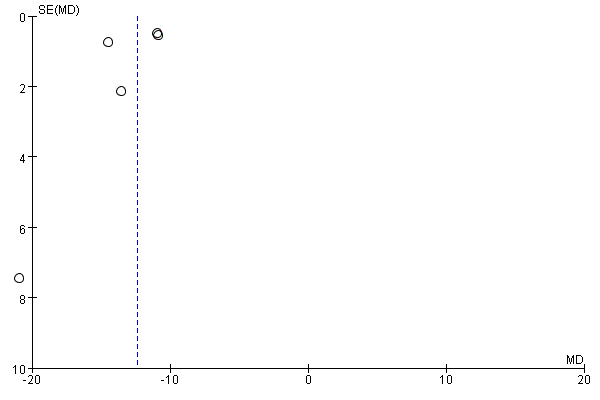


Figure 7. Funnel plot of the amount of analgesics used.


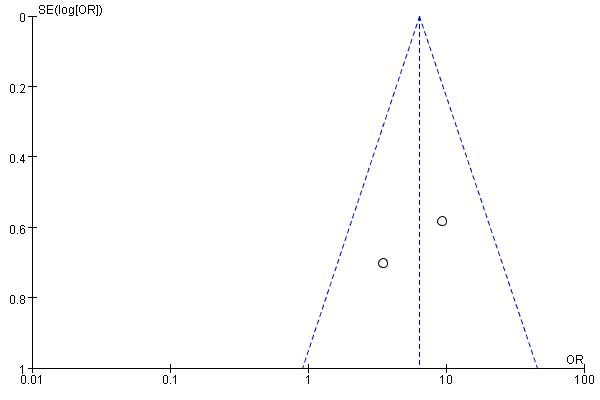


Figure 8. Funnel plot of effective rate.


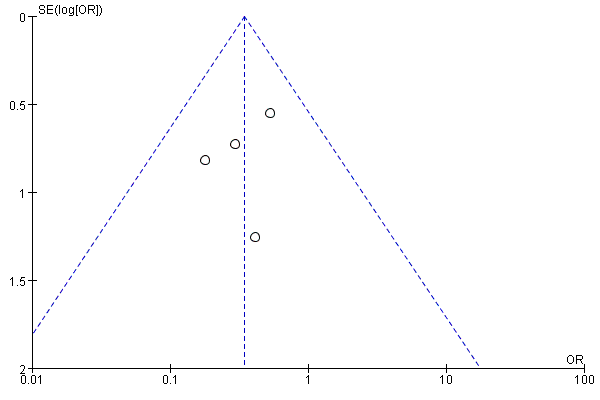


Figure 9. Funnel plot of adverse events.
